# Thoracic Ultrasound: What Non-radiologists Need to Know

**DOI:** 10.1007/s13665-017-0164-1

**Published:** 2017-01-27

**Authors:** Jonathan P. Williamson, Chris Grainge, Ahilan Parameswaran, Scott H. Twaddell

**Affiliations:** 1 Department of Respiratory and Sleep Medicine, Liverpool Hospital, Sydney, Australia; 2 Respiratory, Sleep and Environmental Health Research Group, Ingham Institute for Applied Medical Research, Sydney, Australia; 3 Macquarie University Hospital, Sydney, Australia; 4 Department of Respiratory and Sleep Medicine, John Hunter Hospital, Lookout Road, New Lambton Heights, NSW Australia; 5 Priority Research Centre for Healthy Lungs, Hunter Medical Research Institute, Kookaburra Circuit, New Lambton Heights, NSW Australia; 6 Department of Emergency Medicine, Royal Prince Alfred Hospital, Camperdown, NSW Australia

**Keywords:** Thoracic ultrasound, Lung artefact, Pleural disease, Pleural effusion, Pleural sliding, Seashore sign

## Abstract

**Purpose of review:**

The aim of this review is to provide the theoretical and practical knowledge essential for non-radiologists to develop the skills necessary to apply thoracic ultrasound as an extension of clinical assessment and intervention.

**Recent findings:**

Issues relating to training and competence are discussed and a library of thoracic ultrasound videos is provided to illustrate artefacts, pleural, parenchymal and pneumothorax pathology as well as important pitfalls to consider. Novel and future diagnostic applications of thoracic ultrasound in the setting of acute cardiorespiratory pathology including consolidation, acute interstitial syndromes and pulmonary embolism are explored.

**Summary:**

Thoracic ultrasound requires an understanding of imaging artefact specific to lung and pleura and a working knowledge of machine knobology for image optimisation and interpretation. Ultrasound is a valuable tool for the practicing chest clinician providing diagnostic information for the assessment of pleural and parenchymal disease and increased safety and cost effectiveness of thoracic interventions.

**Electronic supplementary material:**

The online version of this article (doi:10.1007/s13665-017-0164-1) contains supplementary material, which is available to authorized users.

## Introduction

The uptake of thoracic ultrasound (USS) by chest clinicians over the last 10 years represents the most important paradigm shift in procedural pleural medicine since the introduction of fine bore intercostal catheters in the 1980s [1, 2]. As well as guiding thoracentesis, intercostal catheter placement and medical thoracoscopy [3], thoracic USS is valued as an extension of clinical examination in the evaluation of chest pathologies involving pleural diseases [4], parenchymal infections [5, 6] and in the emergent evaluation of the breathless patient [7•, 8]. Whilst thoracic USS has traditionally been the domain of radiologists, non-radiologists have enthusiastically adopted this readily available tool for the management of pleural and parenchymal lung diseases with outcomes similar to those of their radiology colleagues [9, 10].

The potential to cause real harm occasioning death [11, 12] has been the impetus for recent national and international guidelines [13••, 14–16] recommending in the strongest terms, if not mandating, that thoracic USS be taught and used at the bedside prior to attempting invasive thoracic procedures. A growing body of evidence attests to fewer procedure-related complications in addition to cost savings associated with USS use [17–21, 22•].

This article provides a sound theoretical and practical guide for non-radiologist thoracic clinicians to implement point of care USS techniques in the daily management of pleural and parenchymal disease. Other reviews on thoracic USS are already available. However, the real-time nature of USS as a clinical tool is best demonstrated in video rather than still image format and the advent of online journals such as *Current Pulmonology Reports*, provides capacity to utilise short video clips to demonstrate normal artefact and thoracic pathology to further the readers’ understanding.

## Ultrasound Hardware and Knobology

Modern USS machines are much smaller and more portable than in the past making them ideal for use in the emergency department, respiratory wards and procedure suites. The newest generation of scanners feature flat, ‘touch’ keyboards or screens aiming to reduce infection transmission that may occur on conventional keyboards and control panels.

Two probes are commonly used for lung and pleural USS; the large convex and the smaller linear probe. Phased array probes are better suited for cardiac applications and seldom used in thoracic imaging. Size of the probe face is important to ensure close contact with the skin surface whilst fitting in the intercostal space. Generous application of transmission gel is required to ensure sonic coupling into underlying tissue.

Probe selection depends on the resolution and penetration required to view the tissue of interest with higher frequency linear probes providing better resolution but poorer penetration, due to greater attenuation of sound energy. Most clinicians use a linear array probe (with a frequency of around 5–13 MHz and maximum useful imaging depth of approximately 6 cm) to view the pleura as it provides excellent resolution at the short working distances required. These probes are also extremely useful to guide biopsy of chest wall tumours due to the available resolution. Convex probes (with frequency around 1–5 MHz) provide deeper penetration to depths up to 15–18 cm but at the expense of resolution. The curved surface of the convex probe may make it difficult to achieve close skin contact (and therefore sonic coupling) in certain circumstances such as thin patients with prominent ribs.

Understanding USS controls is essential to achieve and maintain a good image. This is often referred to as ‘Knobology’. Although many bedside machines have reduced the number of controls, it is still important to understand their function to best optimise and interpret the images seen.

*Gain* control increases brightness by increasing the amplitude of returning USS waves. Although a bright image may appear to contain more information, too much gain makes an image overly bright and obscures fine detail. *Time gain compensation* (TGC) controls are intended to compensate for the attenuation of sound waves that occurs with depth. TGC is adjusted to make the image brightness more consistent from superficial to deep with each position on the slide corresponding to a discrete zone of the image. Some machines simplify this to include only overall, far-field and near-field gain controls. *Dynamic range*, often not adjustable on more mobile machines, controls the compression of the image with a high dynamic range resulting in lower contrast as the black and white values are brought closer together and more shades of grey are displayed in the image. *Depth* control alters the scanning depth displayed on the screen, whilst the *focal point* of the USS, usually displayed as a triangle or arrow on the side of the image, indicates where maximum resolution occurs. Whilst scanning, we recommend the user frequently adjusts the depth, focus, TGC and overall gain to obtain the best possible image, a task ideally learnt and initially performed under direct supervision of an expert sonographer.

## The Physiology of Lung Artefacts on Ultrasound

An ultrasound probe positioned on a healthy chest wall accurately images through skin, fascia and muscles but detail ends abruptly at the convex cortices of the ribs and between them the pleural line. The high attenuation coefficient of bones [23] results in absorption of around 60% of the delivered USS energy with the remaining 40% reflected back towards the probe. This results in a dark acoustic rib shadow cast distal to the cortical surface beyond which nothing can be imaged (video 1). The pleural line, representing effacement of the parietal and visceral pleura, can be seen between the rib shadows approximately 1 cm below the rib surfaces (video 1). Respiration and cardiac-induced movement of the visceral pleura against the fixed parietal pleura results in lung sliding. The presence of lung sliding excludes a pneumothorax at that site (video’s 1 and 5). High acoustic impedance between the soft tissues *above* and the air-filled lung *below* the pleural line results in reflection of 99.9% of sound energy preventing imaging beyond the pleural line unless soft tissue, fluid or consolidation replaces healthy lung. Hence, in air-filled lung, the USS image deep to the pleural line results entirely from artefact rather than real information from returned USS signal. In the past, these artefacts were thought to make imaging the lung impossible. However, we now use these to infer normal and pathological states of the lung. As such, thoracic USS requires an understanding of the various forms of artefact. In other organ systems, artefact hinders image interpretation and complex algorithms are implemented to minimise their effect such as harmonics, speckle reduction and compound imaging. Where pleura and lung are concerned, artefacts are fundamental to interpretation and these algorithms are best left switched off.


(MPG 17882 kb)


**Fig. 1:**
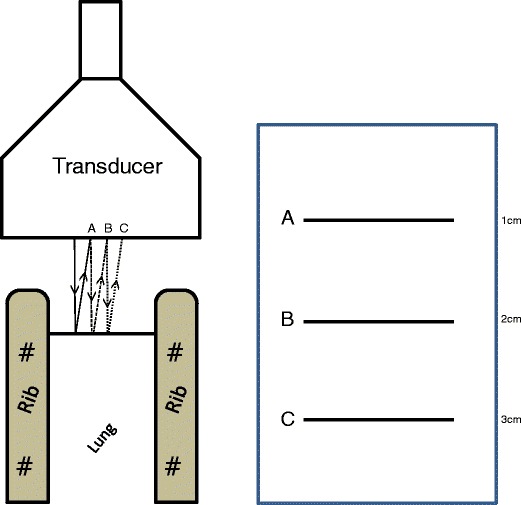
Reverberation artefact caused by the reflection of the sound wave back and forth between the pleural line and transducer results in equidistant A-lines

A-lines are a reverberation artefact producing horizontal, parallel and evenly spaced lines resulting from sound waves reflected back and forth between the ‘mirror-like’ pleural line and the USS transducer (Fig. 1, video 1). In the presence of lung sliding, they indicate a normal visceral and pleural interface.

B-lines (AKA lung rockets), another form of reverberation artefact, are fleeting vertical lines that ring down from the pleural line to the bottom of the US image [24] (video 1). Their aetiology is complex and incompletely understood. The presence of one or two B-lines in a single field of view is normal in healthy individuals, particularly at the lung bases, but more than two B-lines in such a field or their presence throughout the lungs may reflect several pathologies including thickening of the subpleural interlobular septae through fluid (acute interstitial oedema), scarring (interstitial pulmonary fibrosis) and inflammation (infection/atelectasis/consolidation).

Lung comets are short, often transient vertical reverberation artefacts thought to arise from small pockets of fluid between the pleural surfaces (video 1). Unlike B-lines, lung comets do not extend to the bottom of the US image but diminish in intensity over a few millimetres. Lung comets are seen in normal lung and help identify the pleural line.

## The Pathology of Pleural Disease

### Pleural Effusions

The evaluation of pleural fluid, its presence, volume, echogenicity and complexity is perhaps the most common application of thoracic USS. Whilst USS imaging cannot replace biochemical and microbiological assessment of pleural fluid, it does provide clues as to the character of pleural effusion that can be used to assist in determining whether an effusion is likely to be a transudate or exudate.

Four pleural effusion patterns are recognised: anechoic, complex non-septated, complex septated and echogenic. Anechoic effusions are black and featureless on USS (video 2). Transudates are almost invariably anechoic; however, anechoic effusions may be either transudates or exudates. Complex effusions contain small specular reflections from debris within the fluid or areas of increased density. In complex-septated effusions, the development of fibrin stranding results in septations within the fluid dividing it into locules and almost invariably denotes an exudative effusion, often an empyema (video 2). Echogenic regions within effusions may represent tumours, dense collections of pus (empyema), or even atelectatic lung, mimicking solid lesions. It is possible for an empyema (or blood) to be so echogenic that it is not identifiable as an effusion and misinterpreted as lung or a solid lesion (video 6). Therefore, we recommend that any unexpected pleural finding should be carefully evaluated from different angles and in real time to better characterise them.


(MPG 16706 kb)


### Pleural Thickening

Pleural thickening can be present on the visceral or parietal pleural surfaces, seen adjacent to the chest wall or along the diaphragmatic surface when viewed through an effusion. The contrast of pleural fluid against the parietal pleura makes thickening more easily visible. It usually appears as hypoechoic thickening and may be caused by scarring, fibrosis, empyema, pleurisy or tumour. As pleural thickening is hypoechoic, it can easily be misinterpreted as a small pleural effusion. A useful way to help differentiate the two is to evaluate the region with colour Doppler. Free-flowing fluid will produce a signal (“Fluid Colour” sign) due to its movement in response to adjacent lung or cardiac impulse, whereas pleural thickening will be static and produce no Doppler signal [25]. Pleural plaques secondary to asbestos exposure are a relatively common cause of thickening and, if calcified, show focal reflective areas with dense posterior acoustic shadowing. Benign asbestos-related pleural plaques are relatively common; however, other benign pleural masses are uncommon. Although they are usually well-defined and of variable echogenicity, these findings should not be regarded as definitive assessment and other imaging or a biopsy is usually needed to rule out malignancy. Malignant pleural masses include mesothelioma, metastasis from lung tumours (frequently adenocarcinoma) or lymphoma (video 3). Mesothelioma may be nodular or irregular and is frequently associated with a large effusion. Features strongly suggestive of malignant pleural effusion, not necessarily from mesothelioma, include parietal pleural thickening greater than 1 cm, pleural nodularity and diaphragmatic thickening greater than 0.7 cm [4].


(MPG 4640 kb)


## The Pathology of Parenchymal Lung Disease

In addition to the assessment of pleural disease, thoracic USS can evaluate lung pathology including the identification of consolidation and lung abscesses and the localisation of tumours for percutaneous sampling.

### Consolidation

Lung consolidation can only be identified on USS when the area of consolidation extends to the pleural surface; otherwise, as noted earlier, the pleural line forms a barrier impenetrable to USS energy. When lung becomes consolidated, the normally air-filled alveolar spaces become progressively fluid filled, able to transduce sound waves and hence visible on USS. Consolidated lung shows a heterogeneous grey appearance on USS (video 4) usually with irregular or poorly demarcated boundaries. When the consolidation abuts a fluid-filled space (pleural effusion) or an anatomical structure such as the chest wall or diaphragm, then the edge of the consolidated area is well delineated. On plain chest X-ray, air-filled bronchi within consolidated lung are visible as air bronchograms; on thoracic USS, these interfaces between fluid- and air-filled space result in artefact represented as dense white short lines, often projecting distal shadows. These can be followed over time in three dimensions as branching structures termed dynamic air bronchograms (video 4). When power or colour Doppler is used on an area of consolidated lung, the blood vessels can often be traced throughout the consolidated lung. The appearances of consolidated lung on USS mimic those of the liver, and the term hepatisation is used (video 4). As such, it is vital that the liver is separately identified with a delineating diaphragm separating the two structures. It is important to recognise that the highly reflective diaphragmatic surface may produce a mirror artefact of the liver seemingly reflected *above* the diaphragm (video 6). This should not be mistaken for lung consolidation or a highly exudative effusion. Provided the abnormalities reach the pleural surface, thoracic USS is more sensitive than either traditional bedside examination or conventional CXR in diagnosing parenchymal consolidation [26].


(MPG 20632 kb)


### Tumours and Lung Abscesses

Benign and malignant tumours of the lung can be visualised by USS providing they abut the pleura. Round or ovoid uniformly hypoechoic structures on USS are likely to represent tumour, though hyperechoic lesions may be seen. A purely hypoechoic, well-circumscribed mass is most suggestive of a cyst from pleural, bronchogenic or rarely a parasitic origin. In areas of tissue necrosis, whether that be due to necrotic tumour or lung abscess, a fluid density hypoechoic lesion is seen often with a hyperechoic wall and USS reflective ‘debris’ visible within the lesion. Localisation of lung tumours can be helpful for guiding biopsies, with USS comparable to CT in terms of sampling accuracy for pleural or peripheral lung lesions [27, 28]. In addition, thoracic USS may be better than CT to assess chest wall invasion of tumour pre-operatively [29] as well as being able to apply elastography to determine the elastic properties of a lung nodule which may in the future be able to differentiate malignant from benign lesions [30].

### Pneumothorax

Sliding between the visceral and parietal pleura during respiration can be seen on USS in the high reflectivity (white) lines present at the pleura (video 1). In addition to this, A- and B-lines (as noted above) are seen to move laterally with respiration. The use of M mode, where the USS return from a single line of USS energy is displayed through time, highlights this movement visibly and has been described as a ‘seashore sign’ where the movement of the visceral pleura results in lateral shift of the lung artefacts (video 5). Tissues above the pleural line remain relatively static during respiration producing parallel lines on M mode—described as ‘waves’, whereas below the pleural line, artefact appears speckled, like ‘sand’. With a pneumothorax, the parallel lines are continued below the pleural line giving rise to the ‘barcode’ appearance on M mode (video 5). Lastly, power Doppler can be used to view movement of the visceral pleura. The presence of visceral sliding, the ‘seashore sign’ or power Doppler movement enables pneumothorax to be ruled out in most circumstances. This could suggest logically that the absence of these signs ‘rules in’ pneumothorax. However, this is not the case as other pathology resulting in the absence of pleural movement leads to the same findings. Such pathology includes pleurodesis whether therapeutic or spontaneous, hyperinflated lungs with minimal pleural movement as in COPD, large bullae deep to the USS probe, obesity, fibrothorax, pulmonary fibrosis and diaphragm paralysis [31]. In the emergent setting, incorrect intubation of the right main bronchus leading to left lung collapse may also result in absent pleural sliding. Conversely, pneumothorax may be incorrectly ‘ruled out’ when bowel wall or pericardium is mistaken for pleura, or when chest wall movement due to respiratory effort is interpreted as pleural sliding (video 6). USS features of pneumothorax are best seen in the most superior aspect of the chest wall and USS in the supine patient is more sensitive than CXR to detect pneumothorax. Depending on the size of the pneumothorax, there may be a site where moving lung remains in direct contact with the parietal pleura during inspiration, but falls away during expiration. When captured on USS, this is referred to as to the ‘lung point’ sign where the presence and absence of a pneumothorax occur at the same point depending on the phase of the respiratory cycle (video 5). It must be remembered that although lung sliding precludes pneumothorax, the absence of lung sliding does not mean that a pneumothorax is present, [31]. Also, the presence of lung sliding does not exclude a significant pneumothorax elsewhere in the same lung. In our experience, lung sliding on USS does not guarantee that the lung will drop away completely allowing safe access to the pleural space, even locally, when a pneumothorax is induced therapeutically, as is safe to do prior to pleuroscopy [32].


(MPG 16074 kb)



(MPG 19892 kb)


The sensitivity of thoracic USS for the identification of pneumothorax is less than its specificity (sensitivity in the order of 90%, specificity 98%); in the circumstances of a rule out test in a supine trauma patient, it is more sensitive than CXR [33]. The danger comes when the absence of lung sliding, the ‘seashore sign’ or power Doppler are assumed to be pneumothorax, and intervention wrongly performed on that basis.

## A Practical Approach to Bedside Thoracic Ultrasound

A suggested schema for the approach to bedside thoracic USS is shown in Table 1. When setting up for USS, it is important to consider the position of the patient, the operator, the area to be viewed and how long the procedure is expected to take. There will be obvious differences between the use of thoracic USS in trauma and a planned thoracic procedure. For thoracic procedures, the patient should be positioned comfortably with adequate support to maintain a consistent position for any subsequent drainage or biopsy procedure. The operator should also consider their own comfort and ensure proper posture and ergonomics in order to prevent overuse problems, such as chronic shoulder injuries, a common complaint among sonographers [34]. The operator should also consider whether they wish to save images or video to include in the patient record or maintain a procedural log and that these controls, as well as image optimisation controls are within easy reach.

**Table 1:** An approach to bedside thoracic ultrasound

The five P’s then depth-focus-TGC	
Power	Turn on the ultrasound unit
Patient	Ensure patient demographics are entered to ensure images can be saved
Position	Position the patient and ensure a comfortable position for the operator where the screen can be viewed easily
Probe	Select probe—linear, curved linear or phased array
Preset	Select preset—lung or pleural or abdomen
Depth	Set depth
Focus	Set focus
TGC	Set TGC

*TGC* time gain compensation

The transducer probe should be selected based on the anticipated depth and character of the target tissue and any planned procedure. The machine may have a preset function for lung or pleura, but if not, an abdominal setting may be used for deep penetration of USS into an effusion. Next, the depth of the target tissue should be considered. It is usual for the deepest part of the target to be positioned at about three quarters of the maximum depth shown on the screen as this allows the image to be optimised using focus and gain controls. Focus should then be adjusted to optimise the region of interest, usually in the middle third of the image. Finally, the TGC controls should be set to ensure an even gain across the image. Image optimisation is a dynamic process that should be continually addressed throughout the scan to maximise image quality and avoid pitfalls in interpretation (video 6).

It is common to commence scanning on the side opposite the pathology and for the operator to orientate themselves using clearly identifiable landmarks such as kidney, liver, spleen or diaphragm. Especially for the beginner, this helps orientate the sonographer and when unexpected findings are encountered, relative tissue densities may be compared with known organ appearances. It is important to remember that USS provides a two-dimensional representation of a three-dimensional object, and USS planes at 90° to each other should be used to evaluate tissue throughout the scan. It is common to mark a position on the patient’s skin overlying the safest area to pass a needle for biopsy or drainage and once this has been done, the position should be re-checked, again in two planes, prior to any intervention.

At the completion of a scan, it is important to clean the probe and machine appropriately, and although alcohol-containing wipes can be used on the hard surfaces on the machine and probe, a non-alcohol containing cleaner should be used on the transducer surface of the probe to avoid degrading the soft silicon material. There are few risks associated with the use of USS but the potential for cross-infection via probes and USS gel is significant.

## Training Requirements

Whilst there is generally an agreement about the importance of clinician-performed ultrasound for thoracic applications, its use ‘remains highly operator-dependent in spite of advances in technology, and the interests of the patient are best served by the provision of an USS service which offers the maximum clinical benefit and optimal use of resources, i.e. with appropriately trained personnel using equipment of appropriate quality’ [35].

Training in thoracic USS is available at various levels in Europe, North America and the Asia Pacific. A typical 1- to 2-day workshop offers lectures in basic US physics, thoracic anatomy, pleural and parenchymal pathology, supervised hands on practice using simple and/or high fidelity phantoms as well as practice on patients with a variety of pleural pathologies. Some courses also cover different approaches to USS-guided needle aspiration and drain insertion. These are generally viewed as introductory courses and designed to be followed by supervised implementation and skill acquisition at the point of care, with assessment during or after completion of a logbook of cases performed. However, attending a course and keeping a logbook does not equate to competence, and measures of competence assessment are required to confirm adequacy of crucial skills such as insertion site for accessing the pleural space. Recently, thoracic USS assessment tools have been developed to assist in the determination of competence. The UG-STAT is an 11-point assessment task that tests basic USS acquisition skills, image interpretation and localisation of safe ICC insertion sites on either phantoms or real patients and can reliably differentiate novice, intermediate and expert users [36••]. Vetrugno et al. developed an assessment task combining pleural USS with ICC insertion technique in phantoms showing significant differences between novice and expert groups in the critical care setting [37].

Many professional societies and institutions provide accreditation pathways and programs for clinicians in the use of thoracic USS. In the UK, the Royal College of Radiologists in conjunction with the physicians training board mandate a user-directed pathway involving attendance at an accredited course, supervised scanning sessions (minimum 35 scans), a procedure logbook and a competence sheet completed. A novel structured program recently implemented in Australia and New Zealand requires attendance at an accredited course followed by close point of care supervision of USS procedures and documentation of each scan in a logbook (minimum 40 scans) together with a UG-STAT assessment after 10, 20 and 40 scans, the final assessment being a barrier exam (pass mark 90%) [38].

Moving forward, it is suggested that local professional bodies develop accreditation pathways suited to their regional requirements to ensure that as the uptake of thoracic USS broadens, individuals are provided with the necessary skills and training to ensure competence is achieved.

## Future Directions

The benefits of USS have been well described in the management of pleural effusion, particularly in reducing the rate of iatrogenic pneumothorax from thoracocentesis. Studies have shown that the rate of pneumothorax can be reduced by up to 19% through the use of USS [22•]. Bleeding complications from thoracocentesis are less common [39]. Abnormal haemostasis has traditionally been considered a risk factor, but recent studies where USS has been used to delineate visceral structures at risk of perforation suggest that this risk may be overstated [39, 40]. Salamonsen et al. have suggested that USS may also be used to identify exposed intercostal arteries vulnerable to injury in order to further reduce risk [41]. Though promising, the accuracy of USS and the impact on rate of complications requires further research.

Other pleural procedures may benefit from USS use. Real-time USS is comparable to CT in sampling accuracy of pleural lesions, with decreased complications and cost [27]. Physician (as opposed to radiologist) performed closed pleural biopsy under real-time ultrasound guidance has high rates of successful diagnostic sampling [42] and has been advocated as a first line investigation after non-diagnostic aspiration of exudative pleural effusions, potentially replacing thoracoscopy [43].

The role of USS is less well defined in consolidation or interstitial syndromes. A recent review of the literature suggests that USS is more sensitive than CXR for detection of consolidation [44]. However, differentiating between potential aetiologies (such as pneumonia and pulmonary embolism) is heavily dependent on clinical evaluation and suspicion. In the case of pulmonary embolism, two meta-analyses showed sensitivities for diagnosis of PE by thoracic ultrasound of 85% or more [45, 46]. However, the high prevalence of pulmonary embolism in these studies suggests that the USS may only be of use in patients with a high pre-test probability of PE where CTPA is unavailable or contraindicated.

Similarly, in the case of interstitial syndrome, B-lines may be seen with a variety of pathologies. In pulmonary oedema, the number of B-lines on USS has been shown to correlate with extravascular lung water [47], and a recent review demonstrated that thoracic ultrasound had the highest positive likelihood ratio for heart failure when compared to clinical examination, ECG and CXR [48]. B-lines may also be seen with pulmonary fibrosis, and their presence has been used to screen for lung involvement in connective tissue diseases [49]. Other pathologies that may cause B-lines include pneumonia, pulmonary contusions and acute respiratory distress syndrome. Whilst various USS findings have been described to aid in discriminating between these conditions [50–52], their accuracy remains problematic.

In both parenchymal and interstitial lung diseases, the expanded use of USS may improve diagnostic accuracy. For example, thoracic USS combined with lower limb venous USS and echocardiographic assessment increased sensitivity for diagnosis of PE [53]. However, the precise role of USS as a screening tool or as a replacement test remains to be firmly established. At present, it is suggested that physician-performed USS be used to answer simple clinical questions as part of a Bayesian diagnostic approach to avoid the risks of incorrect interpretation.

Finally, whilst USS has been available for decades, ongoing technological developments raise the possibility of new applications. The recent advent of handheld machines and smartphone probe attachments potentially allow for USS assessment as part of routine clinical examination. Colli et al. demonstrated that the use of pocket USS in combination with clinical examination obviated the need for further testing in 95% of patients for whom there was a clinical question of pleural effusion [54]. Another pilot study demonstrated that use of pocket USS by a medical student could screen for the presence of left ventricular systolic dysfunction with a similar sensitivity and specificity to an experienced echocardiologist [55]. Computer algorithms have also been developed to analyze USS images for B-lines [56] and pneumothoraces [57]. Whilst further research and development is required, their use may aid accuracy and reproducibility of USS interpretation, particularly for less experienced clinicians.

## Conclusions

Thoracic interventions should no longer be performed without contemporary imaging and ultrasound represents an accurate, available and cost-effective tool to enable such interventions at the bedside by appropriately trained clinicians. A comprehensive understanding of the sound properties of tissue, air and fluid and the way these interact to produce artefacts is essential for image interpretation as is sufficient bedside experience to apply point of care USS to extend the clinical assessment of pleural and parenchymal disease. Training for current and future chest clinicians should be widely accessible and incorporated into respiratory, cardiothoracic and emergency medicine training curricula and assessment of competence encouraged. It is important to remember that clinicians trained in thoracic USS, particularly in the early part of their careers, are unlikely to have acquired the same level of skill as radiologists or more experienced USS-trained colleagues and we would encourage a close collaborative relationship with such individuals to maximise learning and skill acquisition with the ultimate aim of providing optimal patient care.
